# Supplementary material for: A nonlinear correlation measure with applications to gene expression data
Source: PLoS One. 2022 Jun 21;17(6):e0270270. doi: 10.1371/journal.pone.0270270 (PMC9212159; doi:10.1371/journal.pone.0270270)
Supplement: S1 File — (DOCX) [file pone.0270270.s001.docx]

The training of gamma in the RBF of Kc and

the Kc values for six gene pairs in Application 2

- Data in Application 1 and Application 2 have been **standardized**.

1. **Application 1:**
   - TIAM1 vs. IL17A

The minimum of CV ($1.548 \times{10}^{-9}$) is achieved by $\gamma=7.5$.


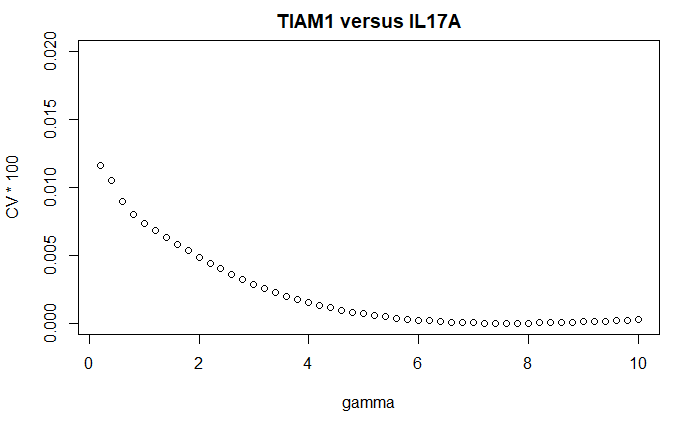


In the following, we show the training of gamma of the RBS kernel in Kc.

- - HST3 vs. RAD51

The minimum of $\mathrm{CV}$ ($1.92 \times{10}^{-8}$) is achieved by $\gamma=4.3$.

But, $CV=7.37\times{10}^{-6}$ when$\gamma=1.0$; the difference between this CV and the min. is $<8 \times{10}^{-6} , so we used Kc-RBF with \gamma=1.0$.


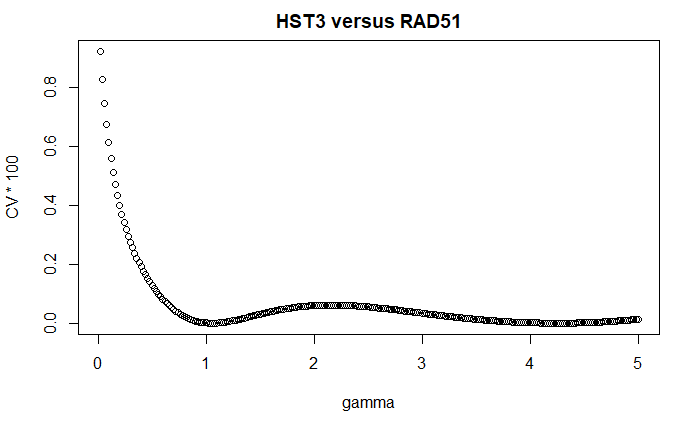


- - HST3 vs. RNR1

The minimum of $\mathrm{CV}$ ($3.74 \times{10}^{-8}$) is achieved by $\gamma=0.9$.

But $CV=7.16\times{10}^{-5} \mathrm{when} \gamma=1.0$; the difference between this CV and the min. is $<8 \times{10}^{-5}, so we used Kc-RBF with \gamma=1.0$.


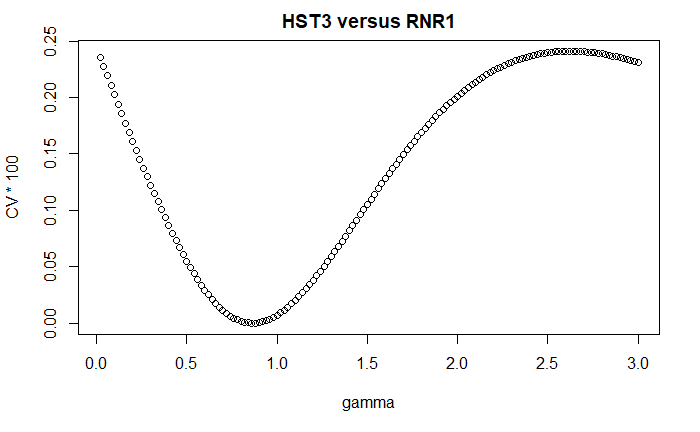


- - HST3 vs. SWE1

The minimum of $\mathrm{CV}$($0.00603$) is achieved by $\gamma=0.7$.


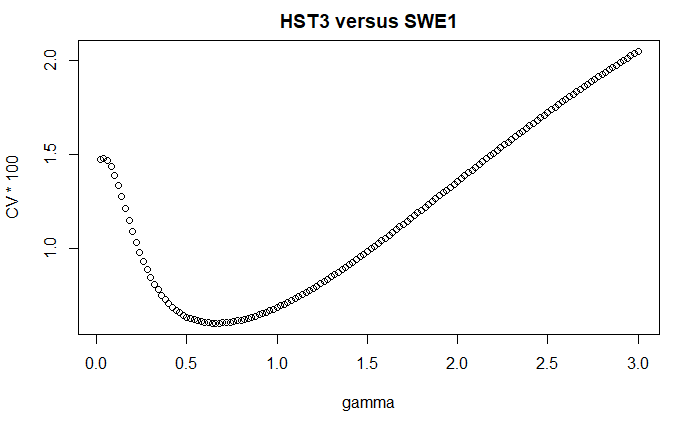


1. **Application 2: the results of Kernelized correlatin (Kc) and Pearson’s correlation**

- For three gene pairs with similar patterns,

Kc-RBF with gamma = 0.5 computed by Repeat 1 and 2 data

| Gene-pairs | Kc (Repeat1) | Kc (Repeat2) | mean | sd | p.value (t-test) |
| --- | --- | --- | --- | --- | --- |
| RNR1-SWE1 | 0.984 | 0.841 | 0.913 | 0.072 | 0.050 |
| RNR1-RAD51 | 0.994 | 0.985 | 0.989 | 0.004 | 0.003 |
| SWE1-RAD51 | 0.865 | 0.953 | 0.909 | 0.044 | 0.031 |

**Pearson correlation results**:

| Gene-pairs | Pearson’s r (Repeat1) | Pearson’s r (Repeat2) | mean | sd | p.value  (t-test) |
| --- | --- | --- | --- | --- | --- |
| RNR1-SWE1 | 0.877 | 0.754 | 0.815 | 0.061 | 0.048 |
| RNR1-RAD51 | 0.883 | 0.954 | 0.918 | 0.035 | 0.024 |
| SWE1-RAD51 | 0.610 | 0.824 | 0.717 | 0.107 | 0.094 |

- **For three anti-similar gene pairs**

Kc with the trained gamma computed by Repeat 1 and 2 data

| Gene-pairs | Kc (Rep1) | Kc (Rep2) | mean | sd | p.value (t-test) |
| --- | --- | --- | --- | --- | --- |
| HST3-RAD51  (RBF, $\gamma=1$) | -0.824 | -0.829 | -0.867 | 0.004 | 0.002 |
| HST3-RNR1  ($RBF, \gamma=1$) | -0.861 | -0.844 | -0.853 | 0.012 | 0.006 |
| HST3-SWE1  ($RBF, \gamma=0.7$) | -0.674 | -0.829 | -0.752 | 0.110 | 0.066 |

**Pearson’s correlation results**:

| Gene-pairs | Pearson’s r (Rep1) | Pearson’s r (Rep2) | mean | sd | p.value (t-test) |
| --- | --- | --- | --- | --- | --- |
| HST3-RAD51 | -0.336 | -0.667 | -0.501 | 0.234 | 0.203 |
| HST3-RNR1 | -0.434 | -0.659 | -0.546 | 0.159 | 0.129 |
| HST3-SWE1 | -0.310 | -0.671 | -0.490 | 0.255 | 0.224 |
