# Supplementary material for: A nonlinear correlation measure with applications to gene expression data
Source: PLoS One. 2022 Jun 21;17(6):e0270270. doi: 10.1371/journal.pone.0270270 (PMC9212159; doi:10.1371/journal.pone.0270270)
Supplement: Attachment — Submitted filename: Response_to_Reviewers.docx [file pone.0270270.s005.docx]

We thank the editor and two reviewers for their helpful comments on our manuscript [PONE-D-21-31352] “A nonlinear correlation measure with applications to gene expression data”. We have revised carefully to address the comments. Please find a list of point-by-point replies below.

**Journal requirements:**

When submitting your revision, we need you to address these additional requirements.

1. Please ensure that your manuscript meets PLOS ONE's style requirements, including those for file naming.

 Reply 1: We have adjusted the title page and the main text according to PLOS ONE style requirements. Changes respond to Reviewers comments/suggestions (changes made by ourselves to improve clarity of the paper) are **marked in blue (in green)** in the file “Revised Manuscript with Track Changes.”

2. We note that the grant information you provided in the ‘Funding Information’ and ‘Financial Disclosure’ sections do not match. When you resubmit, please ensure that you provide the correct grant numbers for the awards you received for your study in the ‘Funding Information’ section.

Reply 2: We’ve revised the “Funding Information” in the Financial Disclosure’ section as follows, and we also included these in the cover letter.

**Financial Disclosure:** The Ministry of Science and Technology, Republic of China, provided funding for this study in the form of grants awarded to author YIC (MOST 108-2118-M-001-004-MY3), to L.S.H. (MOST 107-2118-M-007-002-MY2 and 107-2811-M-007-014 , a postdoctoral fellowship supported SBC), and to GSS (MOST 107-2118-M-001-009-MY2 and 109-2118-M-001-001-MY2). Additionally, Academia Sinica, Taiwan, Republic of China provided a grant to GSS (AS-VTA-110-17). Indirect support was provided through salaries/facilities use by the home institutions of YIC, LSH and GSS. The funders did not have any additional role in the study design, data collection and analysis, decision to publish, or preparation of the manuscript. There was no additional external funding received for this study. The specific roles of these authors are articulated in the ‘author contribution’ section.

3. We note that you have stated that you will provide repository information for your data at acceptance. Should your manuscript be accepted for publication, we will hold it until you provide the relevant accession numbers or DOIs necessary to access your data. If you wish to make changes to your Data Availability statement, please describe these changes in your cover letter and we will update your Data Availability statement to reflect the information you provide.

Reply 3: We have stated the following changes in the cover letter.

**Data Availability:**

“All relevant data are within the paper and its Supporting Information file.”

**Additional Editor Comments:**

Dear Corresponding Author,
according to the referee's comments the paper is accepted for publication.
Best regards.

[Note: HTML markup is below. Please do not edit.]

**Reviewers' comments:**
Reviewer's Responses to Questions

**Comments to the Author**

1. Is the manuscript technically sound, and do the data support the conclusions?

The manuscript must describe a technically sound piece of scientific research with data that supports the conclusions. Experiments must have been conducted rigorously, with appropriate controls, replication, and sample sizes. The conclusions must be drawn appropriately based on the data presented.

Reviewer #1: Yes

Reviewer #2: Yes

2. Has the statistical analysis been performed appropriately and rigorously?

Reviewer #1: Yes

Reviewer #2: Yes

3. Have the authors made all data underlying the findings in their manuscript fully available?

Reviewer #1: Yes

Reviewer #2: Yes

4. Is the manuscript presented in an intelligible fashion and written in standard English?

PLOS ONE does not copyedit accepted manuscripts, so the language in submitted articles must be clear, correct, and unambiguous. Any typographical or grammatical errors should be corrected at revision, so please note any specific errors here.

Reviewer #1: Yes

Reviewer #2: Yes

**5. Review Comments to the Author**

Reviewer #1: The manuscript presents a novel approach to reveal nonlinear correlation in biomedical datasets.
The nonlinear kernel measure has been proposed to project high-dimensional data into Hilbert space. The authors demonstrated the effectiveness their approach for discovery of genes involved in differentiation human T helper cells. The statistical analysis provides sufficient evidence for advantages of the approach with respect to several canonical correlation measures.

Reply to Reviewer #1: We thank Reviewer 1’s careful reading and the kind words.

Reviewer #2: The authors proposed a kernelized correlation coefficient to measure a possibly nonlinear association among genes. Although the proposed method is straightforward, it has potential and broad applicability in bioinformatics. My specific comments are given in the following.

- One obvious competitor is distance correlation to measure the nonlinear association between two variables. So I suggest adding this method to the comparison.

Reply to Reviewer #2: Thank you very much for the suggestions. We have added distance correlation (dCor henceforth) to the comparison in the Simulation study (pp. 12-13 and 14-16) and Applications (pp. 19-22); these and other related changes are marked in blue in the revised manuscript. We have also made changes (marked in green) to improve the clarity of the article.

- I believe the choice of the kernel is quite essential, especially in the applications in genetics. I suggest providing more details about how to choose the kernel in practice.

Reply: Some general rules for kernel selection of Kc are provided on p. 22 and marked in blue.
